# Allelic Exclusion of TCR α-Chains upon Severe Restriction of Vα Repertoire

**DOI:** 10.1371/journal.pone.0114320

**Published:** 2014-12-12

**Authors:** Vasily Rybakin, Luise Westernberg, Guo Fu, Hee-Ok Kim, Jeanette Ampudia, Karsten Sauer, Nicholas R. J. Gascoigne

**Affiliations:** 1 Department of Microbiology, Yong Loo Lin School of Medicine, National University of Singapore, 5 Science Drive 2, Singapore 117545, Singapore; 2 Department of Immunology and Microbial Science, The Scripps Research Institute, 10550 North Torrey Pines Road, La Jolla, CA 92037, United States of America; 3 Department of Cell Biology, The Scripps Research Institute, 10550 North Torrey Pines Road, La Jolla, CA 92037, United States of America; New York University, United States of America

## Abstract

Development of thymocytes through the positive selection checkpoint requires the rearrangement and expression of a suitable T cell receptor (TCR) α-chain that can pair with the already-expressed β-chain to make a TCR that is selectable. That is, it must have sufficient affinity for self MHC-peptide to induce the signals required for differentiation, but not too strong so as to induce cell death. Because both alleles of the α-chain continue to rearrange until a positively-selectable heterodimer is formed, thymocytes and T cells can in principle express dual α-chains. However, cell-surface expression of two TCRs is comparatively rare in mature T cells because of post-transcriptional regulatory mechanisms termed “phenotypic allelic exclusion”. We produced mice transgenic for a rearranged β-chain and for two unrearranged α-chains on a genetic background where endogenous α-chains could not be rearranged. Both Vα3.2 and Vα2 containing α-chains were efficiently positively selected, to the extent that a population of dual α-chain-bearing cells was not distinguishable from single α-chain-expressors. Surprisingly, Vα3.2-expressing cells were much more frequent than the Vα2 transgene-expressing cells, even though this Vα3.2-Vβ5 combination can reconstitute a known selectable TCR. In accord with previous work on the Vα3 repertoire, T cells bearing Vα3.2 expressed from the rearranged minilocus were predominantly selected into the CD8^+^ T cell subpopulation. Because of the dominance of Vα3.2 expression over Vα2 expressed from the miniloci, the peripheral T cell population was predominantly CD8^+^ cells.

## Introduction

Allelic exclusion of T cell receptor (TCR) genes is regulated differently for the α and β-chains [1]–[3]: for the β-chain, rearrangement stops when the cell detects a productively rearranged membrane-bound β-chain protein associated with pre-Tα, leading to downregulation of *Rag1/2* gene expression. Thus only one of the β-chain loci is capable of producing full-length, correctly rearranged, β-chain mRNA and therefore protein. In contrast, the TCR α-chain gene does not cease rearranging until the developing T cell undergoes positive selection. During the CD4^+^8^+^ “double positive” (DP) stage of thymocyte development, both of the α-chain alleles rearrange until a positively-selectable heterodimer is formed with the previously-formed β-chain [4], leading to *Rag1/2*-turnoff which stops further rearrangement [5]–[7]. Immature thymocytes (DP, TCR^lo^) frequently express dual α-chains on the cell surface, but almost all mature (DP, or SP, TCR^hi^) thymocytes express a single αβ-combination [8], [9], in what has been termed phenotypic allelic exclusion [1], [10].

Similarly, most peripheral T cells express a single α-chain on the cell surface, despite frequently (20–30%) having two functionally rearranged and expressed α-chain genes. Estimates of the number of peripheral T cells expressing two cell surface α-chains vary widely from <5% to 15% [10]-[13] in mice, and 30% in humans [14]. The dual receptor cells can cause autoimmunity in some systems [15], [16] or be highly alloreactive [17], although other reports did not find them to increase susceptibility to autoimmunity [11], [18]. They have also been reported to usefully increase the TCR repertoire [19].

A post-translational mechanism ensures that only one α-chain is generally present on the cell surface of mature T cells [1], [9], [10], [20]. This is caused by selective lack of expression of one of the α-chains on the cell surface [9], [10]. This mechanism also operates in transgenic mice expressing two αβ TCRs [21], [22], where it can also regulate β-chain expression [23]. Several mechanisms were proposed to account for this phenomenon, including competition between the α-chains for the β-chain, and “selective retention” of the selectable α-chain on the cell surface [1], [8], [9]. Support for the selective retention model and against α-chain competition models has been obtained [24].

The comparative rarity of cells expressing any individual TCR Vα-region and the lack of suitable reagents, have made it difficult to study TCR α-chain allelic exclusion in thymocytes. Specifically, there are still only mAbs against four mouse Vα-regions: the Vα2 family, Vα3.2, Vα11.1/11.2, and some members of the Vα8 family. There is no anti-Cα mAb that can be used to stain live cells for flow cytometry, and no allelic differences between the Cα-regions of different mouse strains. We therefore decided to make a mouse that can express a diverse repertoire limited to two Vα-regions, as a tool to allow us to study phenotypic allelic exclusion in the thymus. The ultimate goal of this project was to create a mouse model for allelic inclusion of TCR α-chains, which would permit isolation of sufficient numbers of dual α-chain expressing cells for biochemical and cell biological analysis of posttranslational events resulting in the selective retention of a single α-chain.

Using Vα3.2 and Vα2 miniloci, each with two different J-region elements, we found that both the Vα3.2 and Vα2 containing α-chains had a strong ability to be positively selected with either a rearranged Vβ5-containing transgene or the natural repertoire of TCR β-chains. As a result, we were unable to find significant numbers of cells that had rearranged and expressed both minigenes, and were therefore unable to detect significant phenotypic allelic exclusion. We did however find that T cells with the Vα3.2 minilocus-derived α-chain repertoire dominated the Vα2-bearing cells in number. Presumably because of the natural ability of Vα3.2 to skew development of CD8 T cells [25]–[27], this also selected strongly for a CD8^+^ T cell-dominated repertoire.

## Materials and Methods

### Ethics statement

Animal work was performed at TSRI and was approved by the Institutional animal care and use committee of TSRI (protocol #06-0340).

### Mice

C57BL/6 (B6) (CD45.2), B6.SJL-*Ptprc^a^ Pep3^b^*/BoyJ (CD45.1), and MHC^o/o^ β2-microglobulin^−/−^, Aβ^b−/−^, CD45.2) mice were bred and maintained at the TSRI animal facility. Vα2 minilocus mouse was obtained from M. Correia-Neves (Life and Health Sciences Research Institute (ICVS), Braga, Portugal), D. Mathis, and C. Benoist (Harvard Medical School). The Vα3.2 minilocus mouse (Va3Var) was made at TSRI. (*Tcra* ^−/−^) were originally obtained from Jackson labs. The Vβ5 TCR transgenic line was obtained from Dr Pamela Fink, (University of Washington). Vα2 Vα3.2 Vβ5 *Tcra* ^−/−^ mice are herein referred to as “triple-transgenic”.

### Antibodies and flow cytometry

Antibody against Vα2 (clone B20.1) [28] was from eBioscience. Antibodies against Vα3.2 (clone RR3-16) [29] and Vβ5 (clone MR9-4) were from BD Biosciences. Cells were analyzed using LSRII flow cytometer (BD).

### Bone marrow reconstitution

Bone marrow cells were isolated from donor mice, and 2×10^6^ bone marrow cells were injected i.v. into lethally irradiated (11 Gy in two equally split doses) CD45.1 and MHC^o/o^ recipient mice. Thymocytes were analyzed 8 weeks post-reconstitution.

### OP9-DL1 co-culture system

OP9-DL1 cells ([30], [31], kind gift from Dr. J.C. Zúñiga-Pflücker) were seeded at 8,000 cells/well in 24 well plates and cultured in alpha-MEM supplemented with 15% fetal calf serum for 24 hrs prior to thymocyte seeding. Thymocytes were depleted using biotinylated anti-CD4/CD8/CD3 and Lineage Cocktail (CD11c, CD11b, CD19, Ter119, DX5, Gr1, Gl3), followed by anti-biotin beads and passage through Miltenyi LS columns. Remaining immature DN thymocytes were added (80,000 cells per well) to the OP9-DL1 cultures, and incubated as before with the addition of 1 ng/ml of IL-7 (Preprotech). Cells were analyzed 5 days later.

## Results and Discussion

### Transgenic TCR Vα3.2 minilocus

In order to produce a mouse with the ability to make two independent TCR α-chains to allow us to study allelic exclusion, we made use of a TCR α-chain minilocus transgene that allows a Vα2 gene to recombine with Jα26 or Jα2 [32]. Specifically, this is the Vα2.3 (TRAV14-1) variable region exon, which is the same Vα gene used in the MHC class I-restricted OT-I TCR [33]. When bred to the α-chain knockout (*Tcra* ^−/−^) background, this mouse (called “Va2Var”) makes a very limited TCRα repertoire, but with a good degree of diversity in CDR3α [32]. The minilocus construct is based on an earlier vector for making TCR transgenes that uses a natural Vα-promoter [34], such that the transgene is rearranged and expressed at the correct developmental stage. The Vα2 minilocus-derived TCR is expressed in both CD4 and CD8 subsets, with a slight bias to CD4s, although when bred to the Vβ5-containing OT-I TCR β-chain transgene, its expression is skewed to the CD8 cells [32].

We replaced the Vα2 gene with a Vα3 gene (specifically Vα3.2 (TRAV9D-4)) flanked by appropriate recombination signal sequences. With the resulting construct, we made transgenic mice on the B6 background. Vα3.2 was chosen because a good antibody against this V-region exists, and because earlier studies showed that normal Vα3.2 is preferentially expressed in CD8 T cells [25], [27], [29].

We obtained several transgenic founder lines. These were characterized for expression of Vα3.2 and the number of copies of the transgene was estimated by Southern blotting (Table 1). Those with several copies of the transgene (lines #16, 17, and 20) expressed significantly more Vα3.2 in peripheral blood T cells than B6 mice. Line #8 had a single copy of the transgene and expressed about twice the normal amount of Vα3.2 (Table 1). The Line #8 Vα3.2 minilocus mice were bred to the Vα2 minilocus mice on the *Tcra* ^−/−^ background, with or without the Vβ5 OT-I β-chain transgene.

**Table 1:** Expression of Vα3.2 and Vα2 in Vα3.2 minigene transgenic mouse lines.

Line #	Vα3 transgene copy number	% Vα3.2 in CD4	% Vα3.2 in CD8	% Vα2 in CD4	% Vα2 in CD8
Non-transgenic	-	1.2	4.7	13.2	8.5
8	1	2.7	9.3	13.7	8.3
16	∼7	11.5	38.0	12.6	6.1
17	>10	8.9	30.6	13.0	7.1
20	5	5.4	19.5	12.7	7.1

The data in Table 1 also confirmed our previous work showing that Vα3.2 is preferentially selected into the CD8 subset of T cells [25]–[27], as there were about 3.4-fold more Vα3.2^+^ cells in the CD8 subset than the CD4 subset, in each of the Vα3.2 minilocus strains.

### Preferential selection of Vα3.2^+^ T cells over Vα2^+^ T cells

**Figure 1:**
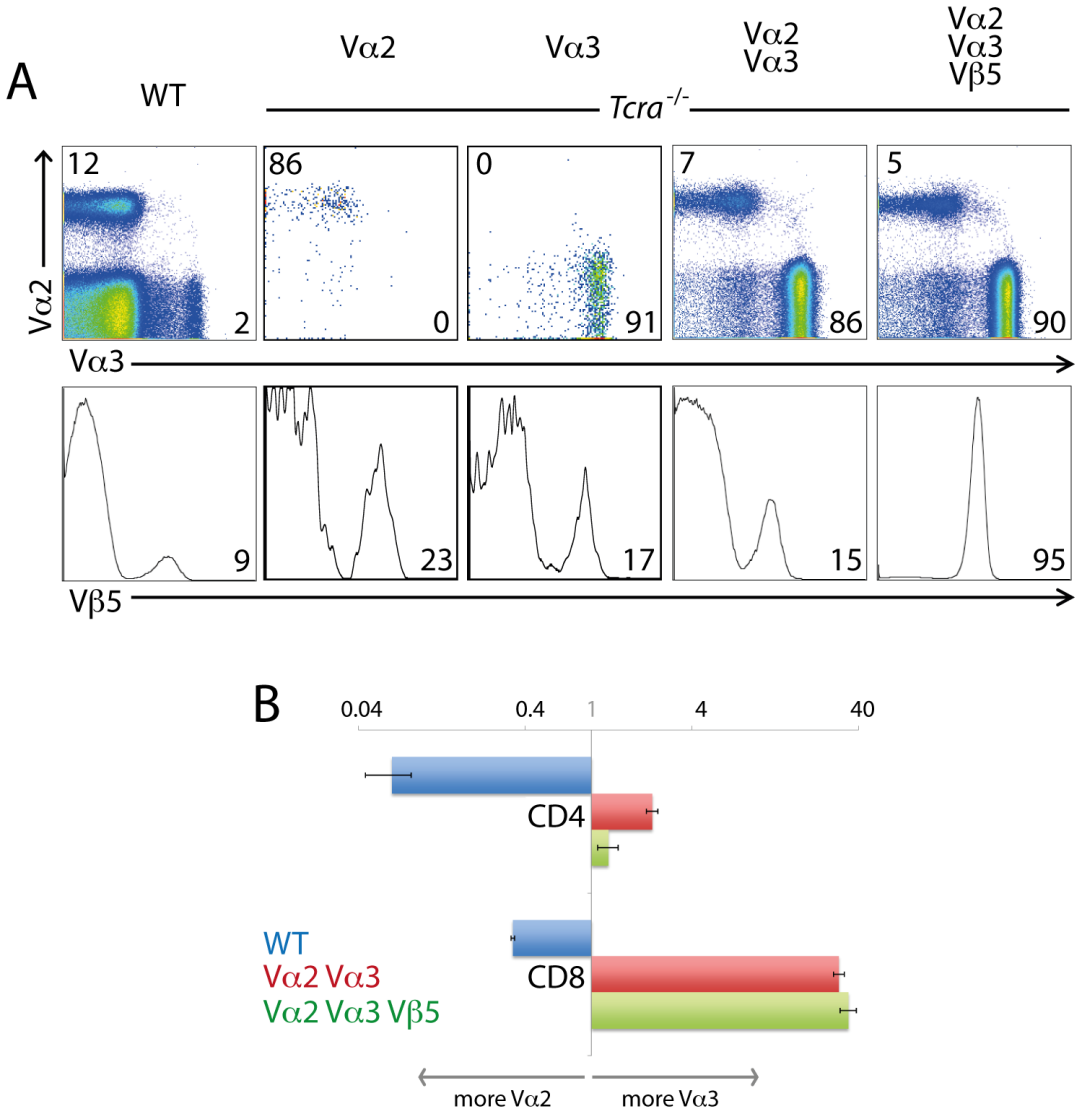
Vα3.2-expressing T cells are more frequent than Vα2-expressing cells in dual α-chain minilocus mice. (A) Vα2, Vα3.2 and Vβ5 expression in lymph node T cells from B6 mice (WT), *Tcra* ^−/−^ mice transgenic for Vα2 or Vα3.2 miniloci, *Tcra* ^−/−^ mice transgenic for both Vα2 and Vα3.2 miniloci, and *Tcra* ^−/−^ mice transgenic for Vα2 and Vα3.2 miniloci plus the rearranged Vβ5 gene. FACS plots representative of >10 mice per genotype. (B) Ratio of Vα3: Vα2-bearing T cells in CD4 and CD8 peripheral T cell subsets in the mice strains described in (A). Data represent >10 mice per genotype.

**Figure 2:**
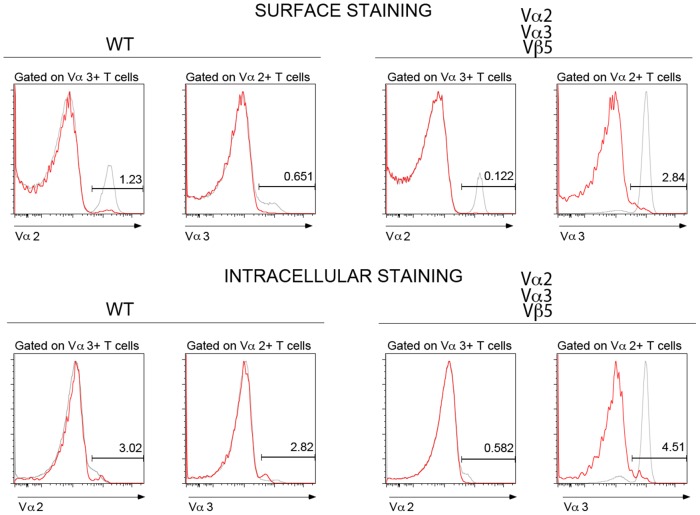
Detection of dual Vα expressing cells in subsets gated based on Vα2-positive or Vα3.2-positive populations. Cells positive for subunits indicated above each plot were analyzed for the expression of the other subunit. Gates were set based on total lymphocyte population (gray lines). Top panel represents detection of dual Vα expressing cells by surface staining, bottom panel by intracellular staining. Results are representative of six mice per genotype.

We tested expression of Vα2, Vα3.2 and Vα5 in mice transgenic for the two Vα miniloci and deficient in endogenous α-chain expression (*Tcra* ^−/−^), and in the presence or absence of the OT-I Vβ5 transgene. In WT B6 mice, Vα2 is expressed in about 12% of T cells, and Vα3.2 in about 2%. However, in the dual minilocus mice, Vα3.2 was much more prevalent than Vα2, whether or not the β-chain transgene was expressed (Fig. 1A,B). Given that the Vα2 minilocus can preferentially reconstitute the OT-I TCR with this β-chain transgene [32], it was a surprise to find such a low representation of the Vα2^+^ cells in the mice expressing the OT-I β-chain. In the WT B6 mice, Vα2 was the more frequently expressed protein, particularly in CD4^+^ cells (Fig. 1B), but the presence of the Vα3.2 minilocus strongly favored Vα3.2 expression, especially among the CD8^+^ cells (Fig. 1B). Analysis of peripheral T cells failed to reveal any significant enrichment of dual Vα expressing cells (Figs. 1 and 2). It was recently noted that the Vα2-Vβ5 combination is disfavored when Vα2 can “choose” a different Vβ element than Vβ5 [35].

**Figure 3:**
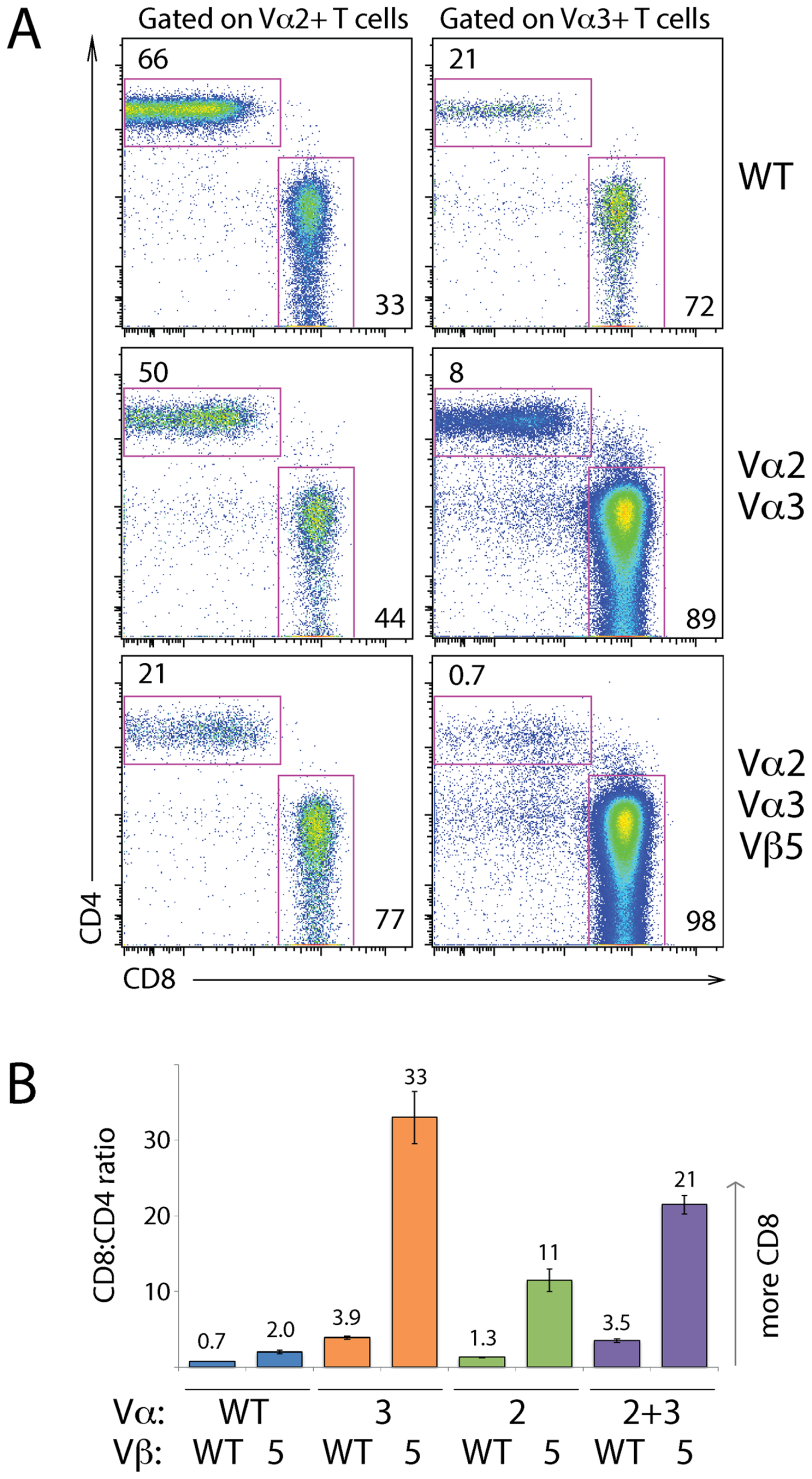
Vβ5 transgene skews expression of Vα2 from CD4 to CD8 subset, but Vα3.2 skews to CD8s with or without Vβ5 transgene. (A) LN cells from B6 mice (WT), *Tcra* ^−/−^ mice transgenic for Vα2 and Vα3.2 miniloci, with or without transgenic rearranged Vβ5, were gated for Vα2 or Vα3.2 expression to show the assortment of cells to CD4 or CD8 subpopulations. FACS plots representative of >10 mice per genotype. (B) shows the CD8∶CD4 ratio of peripheral T cells from mouse strains expressing all the potential combinations of transgenes used here: Vα2 and Vα3.2 miniloci individually or together, and rearranged Vβ5 transgene. Data represent 8–17 mice per genotype.

When lymph node T cells from mice expressing both miniloci as their only α-chains were gated for expression of each of the Vα-regions, the normal CD4-skewing of Vα2^+^ cells was reduced to close to 1∶1 (Fig. 3A, left panels). When the Vβ5 transgene was also present (bottom left panel), skewing was reversed so that the majority of Vα2^+^ cells were CD8^+^. This is as expected from earlier work where Vα2 expression occurred predominantly in the CD4^+^ compartment in B6 mice [28], [36], was roughly equally expressed in CD4^+^ and CD8^+^ T cells in the Vα2 minilocus (Vα2Var) mouse, and was skewed to CD8^+^ cells when the OT-I Vβ5 transgene was coexpressed with the Vα2 minilocus (“Limited mouse”) [32]. Vα3.2 expression in the minilocus mice was even more strongly skewed to the CD8 subset than in WT (Fig. 3A, right panels), and this bias was almost absolute in the double-minilocus, Vβ5 transgene-expressing mice (bottom right panel). These effects were reflected in the CD8∶CD4 ratio (Fig. 3B). This ratio is less than 1∶1 in B6 lymph nodes [36]. The Vα3.2 minilocus caused the ratio to increase to ∼4∶1, but expression of the Vβ5 transgene massively increased the ratio to over 30∶1. Similarly the Vα2 minilocus plus the Vβ5 transgene skewed the population to more than a 10-fold excess of CD8 T cells. This is not surprising given that the OT-I TCR transgene causes development of predominantly CD8^+^ mature T cells, but it is perhaps surprising that the Vα3.2 minilocus had an even stronger impact than the Vα2. This indicates that the potential Vα3-Jα26 and Vα3-Jα2 rearrangements were able to produce very positively selectable TCRs in combination with the OT-I β-chain, just as they were with a complete β-chain repertoire. It also shows the strength of the preferential selection of Vα3.2 into MHC class I-restricted T cells [25], [27].

### Expression of the α-chain miniloci in thymocytes

**Figure 4:**
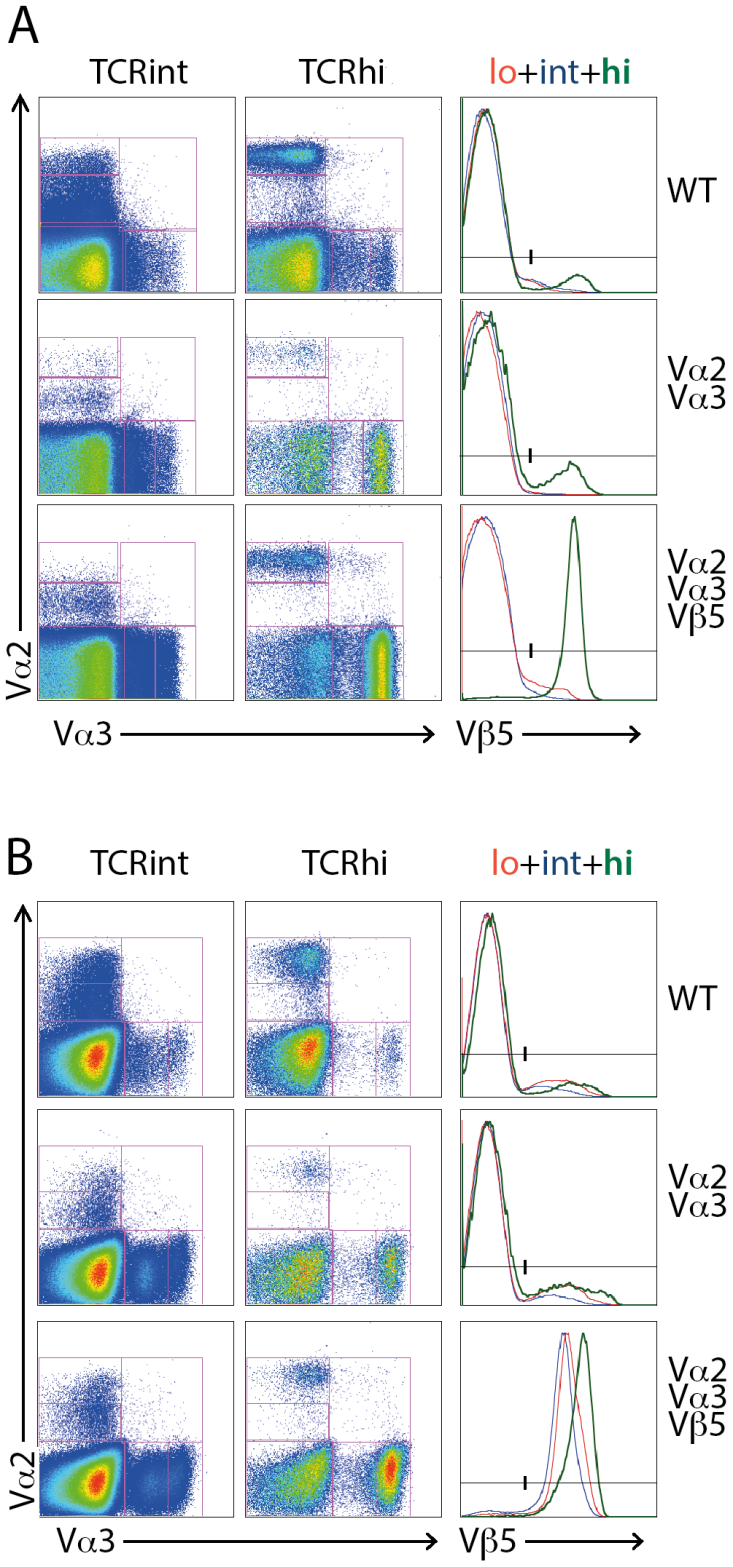
Triple-transgenic thymocytes exhibit efficient allelic exclusion of TCR Vα chains. (A) Double-positive thymocytes from B6 (top), Vα2 Vα3.2 (middle) and Vα2 Vα3.2 Vβ5 (bottom) mice were analyzed for surface expression of Vα2, Vα3.2 and Vβ5 in TCR^int^ and TCR^hi^ gate. (B) Double-positive thymocytes from B6 (top), Vα2 Vα3.2 (middle) and Vα2 Vα3.2 Vβ5 (bottom) mice were analyzed for intracellular expression of Vα2, Vα3.2 and Vβ5 in TCR^int^ and TCR^hi^ gate. FACS plots representative of >10 mice per genotype.

**Figure 5:**
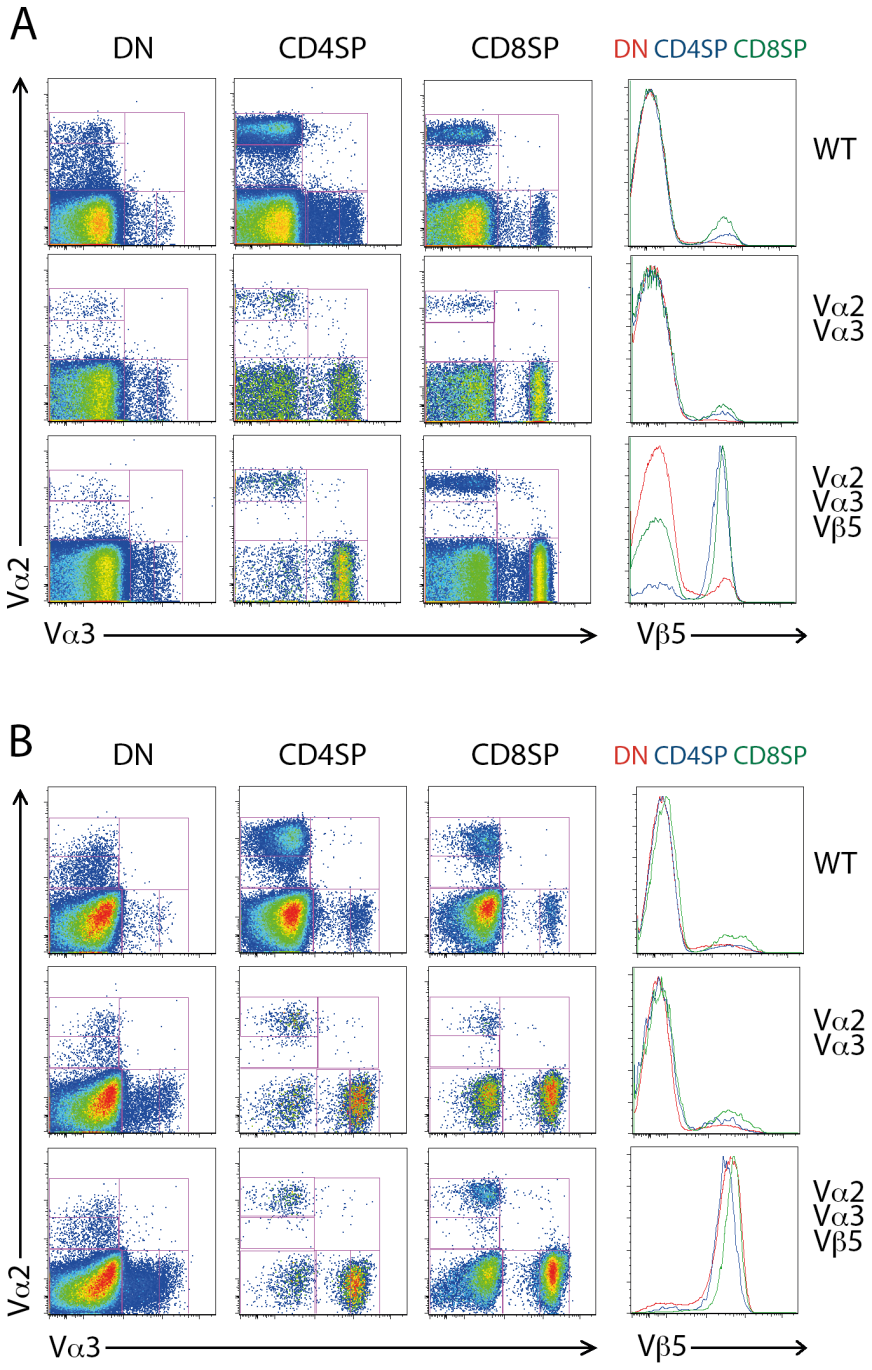
Triple-transgenic thymocytes exhibit efficient allelic exclusion of TCR Vα chains. (A) Double-negative (DN) and CD4 and CD8 single positive (SP) thymocytes from B6 (top), Vα2 Vα3.2 (middle) and Vα2 Vα3.2 Vβ5 (bottom) mice were analyzed for surface expression of Vα2, Vα3.2 and Vβ5 in TCR^int^ and TCR^hi^ gate. (B) DN and CD4 and CD8 SP thymocytes from B6 (top), Vα2 Vα3.2 (middle) and Vα2 Vα3.2 Vβ5 (bottom) mice were analyzed for intracellular expression of Vα2, Vα3.2 and Vβ5 in TCR^int^ and TCR^hi^ gate. FACS plots representative of >10 mice per genotype and are derived from the same dataset as Fig. 4.

We analyzed expression of the two α-chain minilocus transgenes in *Tcra* ^−/−^ thymocytes, finding that, as with the peripheral T cells, the vast majority of thymocytes expressed Vα3.2 rather than Vα2, whether or not Vβ5 was also expressed (Fig. 4A). This was true of the TCR^int^ (before or during positive selection) cells, as well as the TCR^hi^ post-positive selection cells. There was no evidence for increased numbers of dual TCR α-chain-expressing cells compared to WT thymi in any of these developmental subsets. Because we had previously noted that some cells express two α-chains intracellularly, with only one being expressed on the cell surface [10], we also tested for expression of the two transgenes in permeabilized cells. Intracellular expression of either Vα3.2 or Vα2 from the miniloci was found, but there were essentially no cells expressing both Vα3.2 and Vα2 (Fig 4B). Separate analysis of the DN subset and CD4 and CD8 SP subsets did not reveal significantly enriched dual Vα-expressing cells (Fig. 5). Rare dual Vα positive cells were indeed observed in all specimens, however regardless of the phenotype this population represented no more than several hundred cells per mouse.

Because α-chain rearrangement and expression from one chromosome does not inhibit rearrangements on the other chromosome [1]–[3], we were initially surprised that there was not a population of cells expressing both miniloci. However, it was previously found that that only a relatively small proportion (∼0.5% of normal) of the thymocytes in the Vα2 minilocus mice rearrange and express the locus [32]. This suggested that the lack of cells expressing both Vα3.2 and Vα2 was due to the low numbers that actually rearrange either of the miniloci. As noted earlier, both of the miniloci produce highly selectable α-chains, so that the lack of dual Vα-expressing thymocytes could be due to positive selection occurring before there is a chance for rearrangement of the other minilocus.

### Vα expression in thymocytes in the absence of a selecting signal

**Figure 6:**
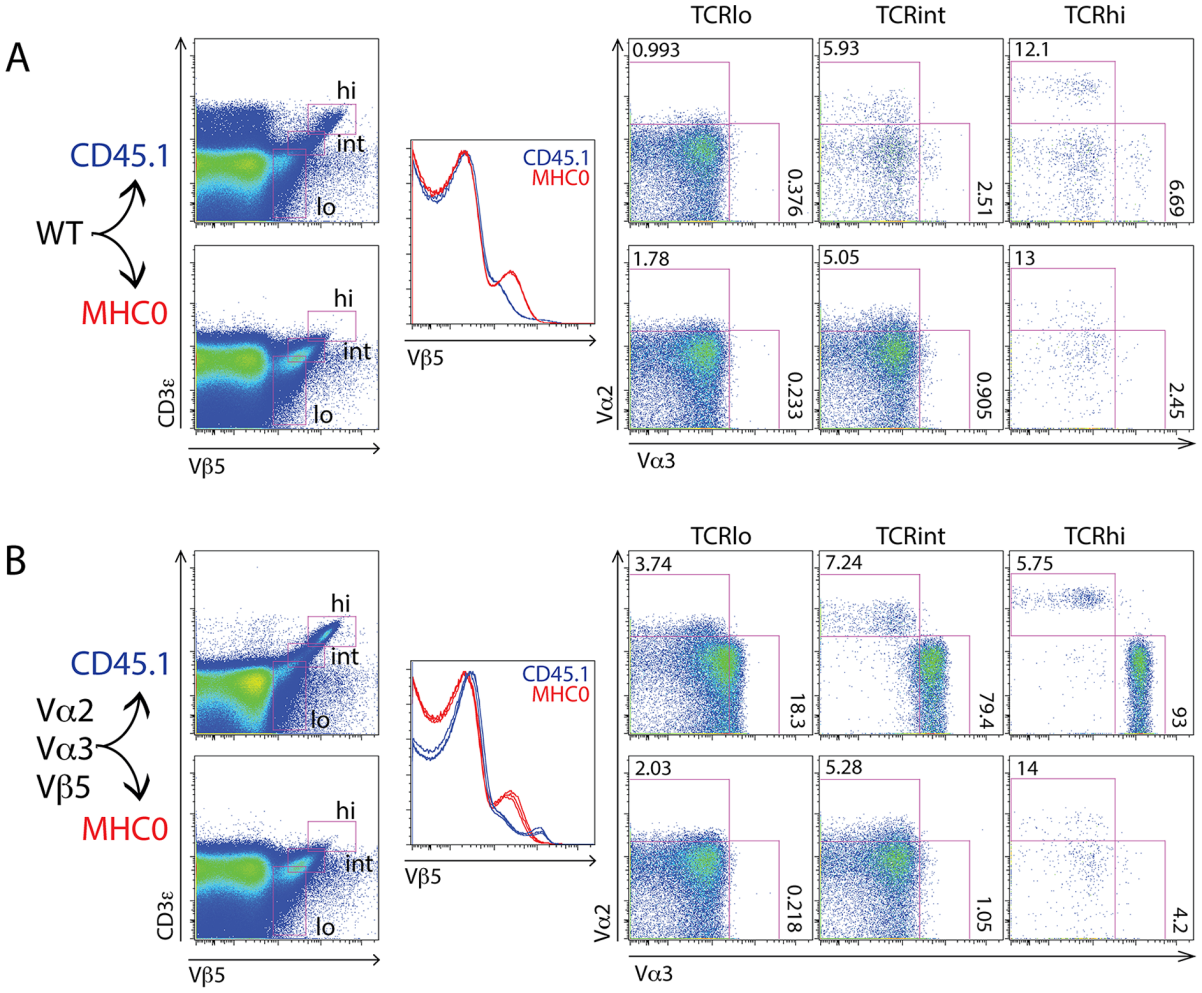
Development of triple-transgenic cells in hosts lacking MHC class I and II yields TCR^int^, single α-chain thymocytes. (A) Bone marrow from B6 mice was used to reconstitute irradiated CD45.1 (top) or MHC^o/o^ (bottom) hosts. After eight weeks, thymocytes were harvested and analyzed for expression of either α-chain in TCR^lo^, TCR^int^, and TCR^hi^, Vβ5-positive gates. (B) Bone marrow from triple-transgenic mice was used to reconstitute irradiated CD45.1 (top) or MHC^o/o^ (bottom) hosts. After eight weeks, thymocytes were harvested and analyzed for expression of either α-chain in TCR^lo^, TCR^int^, and TCR^hi^, Vβ5-positive gates. FACS plots representative of 7-9 mice per group in three independent experiments.

We reasoned that lack of dual Vα-expressing cells might be due to efficient positive selection of both the single-Vα3.2 or single-Vα2-expressing cells, before they had a chance for a second rearrangement. We therefore tested whether the dual minilocus thymocytes could be induced to co-express both Vα2 and Vα3.2 if they were allowed to develop in a non-selecting background. Chimeric mice were made by reconstituting lethally irradiated CD45.1 mice or MHC^o/o^ host mice with bone marrow stem cells from either normal B6 mice or mice with transgenic Vα2 minilocus, Vα3.2 minilocus, and Vβ5 β-chain, on the *Tcra* ^−/−^ background. Eight weeks after reconstitution, the mice were sacrificed and thymocytes analyzed (Fig. 6). In the MHC-sufficient CD45.1 hosts, the transgenic thymocytes developed as in the donor mice, with the vast majority of mature thymocytes expressing Vα3.2 and about 6% expressing Vα2. In the MHC^o/o^ host thymi, very few cells progressed past the TCR^int^ stage, and in most chimeras there were no detectable dual Vα2 and Vα3.2-expressing cells in the TCR^int^ cells (Fig. 6B, lower panel).

**Figure 7:**
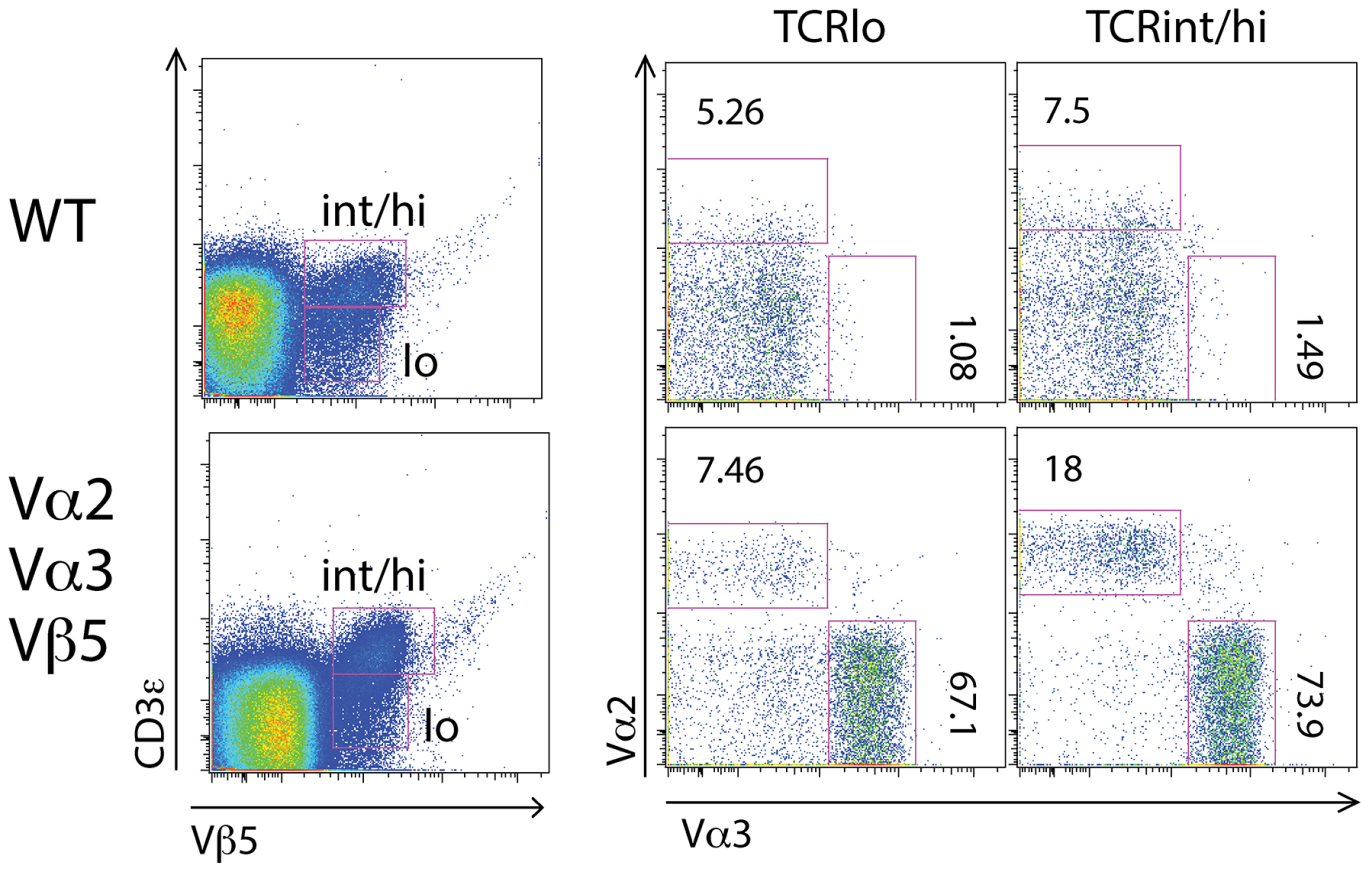
Development of DN thymocytes from triple-transgenic mice in OP9-DL1 in vitro system yields TCR^int/hi^, single α-chain thymocytes. (A) DN thymocytes from B6 mice were enriched as described in Materials and Methods and co-cultured with OP9-DL1 cells in the presence of IL-7 for five days. Cells were harvested and analyzed for expression of either α-chain in TCR^lo^, and TCR^int/hi^, Vβ5-positive gates. (B) DN thymocytes from triple-transgenic mice were enriched and co-cultured with OP9-DL1 cells as above. Cells were harvested and analyzed for expression of either α-chain in TCR^lo^, and TCR^int/hi^, Vβ5-positive gates. FACS plots representative of 4 mice per genotype in triplicate wells and two independent experiments.

In another experiment to test the effect of eliminating positive selection, we used the OP9-DL1 co-culture system [30], [31] to analyze thymocyte development. In this system, thymocytes develop from DN to DP TCR^int^ cells, but few undergo positive selection to produce TCR^hi^ cells. Here, the triple-transgenic (Vα3.2 and Vα2 miniloci, rearranged OT-I Vβ5, *Tcra* ^−/−^) cells developed from DN to DP, expressing TCR^lo^ or TCR^int^ (TCR^hi^ cells were not distinguishable, and were counted with TCR^int^). Again, negligible numbers of dual Vα-expressing cells were found (Fig. 7).

## Conclusions

Our initial goal was to produce a mouse expressing two defined Vα's that would allow us to study the induction of phenotypic allelic exclusion in detail, and particularly biochemically. This goal was not realized, likely because of the low frequency of cells in these transgenic mice that actually rearranged the α-chain miniloci. Moreover, once expressed, both the Vα3.2 and Vα2 containing α-chains were efficiently positively selected. This was the case with endogenously rearranged TCR β-chains as well as when the β-chain repertoire was limited to the OT-I (Vβ5) β-chain. This likely contributed to the failure to find a distinct population of cells that expressed dual TCRs, even intracellularly. Vα3.2 transgene-expressing cells were much more frequent than the Vα2 transgene-expressing cells, especially in the presence of the OT-I β-chain. This was surprising given that the OT-I TCR uses the identical Vα2 element to the minilocus and that the OT-I CDR3α can be recreated by rearrangement of the minilocus [32], although other work also suggests that the combination of Vα2 with Vβ5 is not particularly strongly selected when other Vα-Vβ combinations are possible [35]. The predominant selection of Vα3.2-bearing cells to the CD8^+^ population [25]-[27] was recapitulated in the minilocus mice, and the dominant expression of Vα3.2 compared to Vα2 resulted in the majority of peripheral T cells being CD8^+^ cells. The Vα3.2 minilocus mouse strain will potentially be useful for studies of repertoires using the Vα3.2 element, similar to the utility of the previously-described Vα2 minilocus strain.
